# Development of Fermented Dried Salted Spanish Mackerel and Analysis of Its Flavor Compounds, Fermentation Conditions, and Preparation of a Directed Vat-Set Starter

**DOI:** 10.3390/foods15162862

**Published:** 2026-08-17

**Authors:** Hong Xiao, Ruidi Wang, Yanning Zhao, Haiyan Huang, Gang Yu, Yong Xue, Changhu Xue

**Affiliations:** 1 College of Biological and Food Engineering, Hubei Minzu University, Enshi 445000, China; xiaohong463108359@163.com; 2 Department of Food Science and Engineering, Ocean University of China, Qingdao 266003, China; wrd0186@outlook.com (R.W.); qkz121231@163.com (Y.Z.); m15966945778@163.com (H.H.); xuech@ouc.edu.cn (C.X.); 3 Yantai Marine Economic Research Institute, Yantai 264003, China; 4 Weifang Vocational College, Weifang 262737, China; 5 Key Laboratory of Effcient Utilization and Processing of Marine Fishery Resources of Hainan Province, Sanya Tropical Fisheries Research Institute, Sanya 572018, China; 6 Sanya Tropical Fisheries Research Institute, Sanya 572018, China

**Keywords:** Spanish mackerel, flavor compounds, physicochemical properties, fermentation conditions, directed vat-set starter

## Abstract

In this study, fermented, dried, salted Spanish mackerel (FDSSM) was prepared using a composite fermentation agent, and its flavor compounds, fermentation conditions, and preparation of a directed vat-set starter were investigated. The results showed that adding a combination of *Lactobacillus sakei* (LS), *Pediococcus pentosaceus* (PP), and *Lactobacillus casei* (LC) at a ratio of 1:1:1 yielded FDSSM with the best sensory quality. LS+PP+LC samples exhibited the highest 5′-IMP and HX content at 492.07 and 526.36 mg/100 g, respectively, and the lowest TBARS content at 0.76 mg/100 g. A total of 77 flavor compounds were detected across the five samples. The key flavor compounds in the FDSSM were 3-methylbutanal, *n*-hexanal, *n*-heptanal, *n*-octanal, nonanal, 1-octen-3-ol, and heptanol. The optimal fermentation conditions for FDSSM were a fermentation time of 5.5 h, a fermentation temperature of 28.5 °C, and a strain addition amount of 4%. The optimal composite protectant for the directed vat-set starter was 15% inulin, 5% gum arabic, 5% skim milk powder, and 5% sodium glutamate at a 1:1:1:5 ratio, yielding the highest microbial protection rate of 69.86 ± 0.23%. The optimal storage temperature for the directed vat-set starter was −20 °C. The viable bacterial count in starter cultures stored for 60 days exceeded 10^9^ CFU/g. The addition of a directed vat-set starter yielded FDSSM with superior sensory quality compared with mixed bacterial cultures. These findings provide a theoretical foundation for the large-scale industrial production of FDSSM.

## 1. Introduction

Spanish mackerel, which is primarily distributed in the northwestern Pacific Ocean, is primarily produced in the East China Sea, Yellow Sea, and Bohai Sea, particularly in the southern coastal fishing grounds of Shandong Province, China. Spanish mackerel has soft flesh, only a few bones, and is rich in nutrients, such as vitamins, proteins, calcium and other minerals, and polyunsaturated fatty acids (PUFAs), which benefit physical and mental health [1]. In Shandong, this economically important fish is processed and sold as fresh or cured products, including salted and dried Spanish mackerel. However, at present, the processing of dried salted Spanish mackerel is still dominated by traditional, handmade, workshop-type procedures. Hence, the resulting products still have problems, such as high salt content, unstable flavor, and single taste. Previous studies have investigated the key flavor components and potential metabolic mechanisms of salted dried Spanish mackerel, as well as the effects of *Lactobacillus plantarum* R-1 fermentation on its taste profile [2,3,4,5].

Moreover, the industrialized processing of Spanish mackerel remains seriously limited. Fermentation technology is widely employed in China to produce processed fish products, such as fermented sea bass [6,7], wine lees golden pomfret [8,9], and stinky mandarin fish [10]. Fermented fish often undergo several beneficial biochemical changes as a result of microbial or enzymatic action during acidification, such as myofibrillar and sarcoplasmic protein gelation, as well as protein and lipid breakdown. Muscle protein gelation influences the elasticity, cohesion, and hardness of fish flesh. Furthermore, protein and fat degradation generate compounds that enhance fish digestion and absorption [11]. Therefore, fermentation technology can be applied to study dried salted Spanish mackerel to improve its quality and flavor.

Studies have shown that the dominant bacterial strains in traditional dried and salted fish products are lactic acid bacteria, micrococci, and staphylococci [10,12]. During product processing, the dominant bacteria play an important role in inhibiting the growth of color-, texture-, flavor-, and spoilage-related bacteria [13,14]. Among them, lactic acid bacteria can metabolize carbohydrates to produce aromatic metabolites, such as acetaldehyde and diacetyl, in a microaerobic environment, so that the fermented products have good flavor characteristics [11,15]. Meanwhile, lactic acid bacteria can inhibit the growth and reproduction of spoilage and pathogenic bacteria through acid production and other pathways, playing a decisive role in stabilizing product quality. *Staphylococcus* belongs to the family of micrococcus bacteria, which produce acid slowly. It is usually mixed with lactobacilli to secrete lipolytic enzymes and proteases during the fermentation process, promoting the production of volatile components and enhancing the flavor quality of food [2,11]. Therefore, an increasing number of *Lactobacillus* and *Staphylococcus* species are being used in the production of dry-cured fish products to improve their flavor and texture. However, there are few relevant reports on the study of fermented, dried, salted Spanish mackerel (FDSSM). Specifically, there are no relevant studies on the effects of composite fermentation agents on the quality of salt-dried Spanish mackerel.

Directed vat-set starters are highly viable, highly concentrated, and standardized solid-state fermenters made from lactobacilli after drying and aseptic packaging. Compared to liquid fermenters, directed vat-set starters have advantages of convenient use, transportation, storage, and high fermentation vigor. They can also effectively reduce production costs, shorten the production cycle, and improve the stability and safety of products during production [16]. Directed vat-set starters are widely used in food processing, such as in pickled radishes [16], winemaking [15], and Sichuan Paocai [3]. However, no relevant research has been conducted on directed vat-set starters currently used for fermenting salt-dried Spanish mackerel.

Therefore, the present study aimed to (1) prepare several mixed ferments in different combinations using fermentation strains such as *Lactobacillus sake* and *Staphylococcus xylosus* to develop FDSSM and study the physicochemical properties and flavor compounds of the resulting FDSSM; (2) optimize the fermentation conditions of FDSSM, including fermentation temperature, fermentation time, and the amount of bacterial species added; and (3) prepare a directed vat-set starter of FDSSM. This study serves as a theoretical foundation for the industrial manufacturing of FDSSM.

## 2. Materials and Methods

### 2.1. Materials

Spanish mackerel was sourced commercially from the Nanshan Live Fresh Aquatic Products Wholesale Market (Qingdao, China). Each fish had an average weight and length of 275 ± 25 g and 27.5 ± 2.5 cm, respectively. The fish were transported to the laboratory through a cold chain system and immediately stored in a −20 °C refrigerator for preservation. The edible salts were purchased from Liquin Mart (Qingdao, China). *Lactobacillus sakei* (LS) was isolated and purified from commercially available dried and salted Spanish mackerel. *Lactobacillus acidophilus* (LA) and *Lactobacillus casei* (LC) were preserved at the Laboratory of High Value Utilization Technology of Aquatic Products of the Ocean University of China (Qingdao, China). *Pediococcus pentosaceus* (PP) and *S. xylosus* (SX) were purchased from the China Center for Industrial Culture Collection (Qingdao, China).

### 2.2. Research on the Antagonistic Effects of Different Lactic Acid Bacteria

Research on the antagonistic effects of different lactic acid bacteria was performed as described by Liu et al. (2010) [17]. Briefly, LS was inoculated onto solid MRS medium as an indicator organism. Sterile filter paper discs were then placed flat on the plates and appropriate volumes of other bacterial cultures were added as test organisms. The plates were incubated at 30 °C for 48 h, and the presence of clear zones of inhibition around the filter paper discs was observed. To exclude the effect of acids, the MRS broth was adjusted to pH 3.9 with lactic acid serving as a blank.

### 2.3. Preparation of FDSSM Samples

LS, LA, and LC were sub-cultured twice in MRS broth, while PP and SX were sub-cultured twice in NB broth. Cell pellets were obtained through cryogenic centrifugation (Eppendorf China Limited, Shanghai, China), washed twice with sterile water, and bacterial counts were adjusted to 1 × 10^8^ CFU/mL. Frozen Spanish mackerel was thawed in running water for 1.5 h. The head, tail, internal organs, and vertebrae were removed, and the fish were rinsed with running water. Next, the fish were cut into 5 cm × 5 cm cubes, with each cube weighing approximately 50–60 g, and the excess surface water was removed. The dry pickling method was used, adding 4% (*w*/*w*) salt to pickle at 10 °C for 1 h, and then rinsing under running water to desalt. Based on a preliminary experiment, the fish were divided into four groups using the smear method and adding 4% (*v/w*) of the mixed strains. The LS+PP+LC samples were inoculated with a mixed fermenter ratio of LS:PP:LC = 1:1:1; LS+PP+LC+SX samples were inoculated with a mixed fermenter ratio of LS:PP:LC:SX = 2:2:2:1; PP+LC samples were inoculated with a mixed bacterial suspension containing PP:LC at a ratio of 1:1; and the control was subjected to natural fermentation (no exogenous microorganisms were introduced). Fermentation was conducted at a constant temperature of 23.5 °C for 5.5 h. Cold air-drying was performed for 24 h with a set temperature of 20 °C, a bypass volume of 0.8, and an air velocity of 2 m/s. The samples were then air-dried in cold air-drying mode. Commercially available salted and dried Spanish mackerel samples were used as commercially available (CA) samples along with the other four groups of samples for subsequent assays. Finally, each sample was stored in a refrigerator at −80 °C until further analysis.

### 2.4. Sensory Analysis of FDSSM Samples

The samples were examined and sensorially evaluated using the approach described by Zhao et al. (2016) [18] and Wu et al. (2021) [19], with a few modifications. The FDSSM sensory descriptors completed three 20-min training sessions before the analysis. Twelve volunteers (six females and six males, aged 22–50 years), including graduate students and faculty members from the Ocean University of China who wished to try the FDSSM, were selected for training using questionnaires. Volunteers were chosen based on their ability to describe the texture, aroma, color, acceptability, and taste of the FDSSM samples through examination by touching with chopsticks, smelling, and tasting. The quality of the FDSSM was divided into four categories: 10–8, 7–6, 5–4, and 3–0. The quality features of the FDSSM, including the mouthfeel, aroma, color, acceptability, and taste, were assessed separately. The sensory evaluation results were averaged. Ethics approval for this study was obtained from Hubei Minzu University (approval number: 2026062). All participants were provided with information regarding the study and gave their written informed consent prior to participation. This study was conducted in compliance with the Declaration of Helsinki and all applicable ethical guidelines.

### 2.5. Analysis of the Flavor Nucleotides FDSSM

Flavor nucleotide analysis was performed as described by Bi et al. (2022) [20]. The identity and quantity of nucleotides were determined by comparing the standard retention duration and the peak region of each nucleotide. The standard curve equation was obtained by using the peak area Y and the CMP, IMP, Hx, AMP, and the HxR content (ng/μL) as X, as described in the equations Y = 43.161X + 10.145 (R^2^ = 1.0000), Y = 97.316X + 115.7 (R^2^ = 0.9996), Y = 251.4X + 681.54 (R^2^ = 0.9984), Y = 122.05X + 457.17 (R^2^ = 0.9971) and Y = 136.54X + 318.35 (R^2^ = 0.9991), respectively.

### 2.6. Free Amino Acid Analysis of FDSSM Samples

Crushed skinless Spanish mackerel meat (8 g) was thoroughly mixed with 15 mL of 0.02 M hydrochloric acid. The sample was sonicated for 5 min and then centrifuged at 8520× *g* for 20 min at 4 °C. Free amino acid (FAA) analysis was performed as previously described (Bi et al., 2022) [20].

### 2.7. Texture Profile Analysis (TPA)

A texture analyzer was used to conduct TPA (TMS-Pro, Food Technology, Inc., Shanghai, China). Briefly, Spanish mackerel meat samples were cut into 2 cm × 1.5 cm × 1.5 cm cubes and obtained from identical anatomical areas of the fishes. TPA experiments were performed according to the methodology reported by Xiao et al. (2022) [6].

### 2.8. Gas Chromatography–Mass Spectrometry (GC-MS) Analysis of FDSSM Samples

Crushed skinless Spanish mackerel meat (2 g) was weighed into a 20 mL headspace vial, and then 3 mL of saturated NaCl solution and 60 μL of an internal standard solution (2,4,6-trimethylpyridine, 10 ppm) were added. The vial was then sealed and placed in a shaking incubator at 60 °C for 30 min at 500 rpm. Samples were extracted using a solid-phase microextraction (SPME) needle for 30 min at the same temperature. Immediately after extraction, the SPME extraction head was inserted into the GC injection port and resolved at 250 °C for 5 min. GC-MS analysis was performed as previously described by Xiao et al. (2023) [7].

### 2.9. Determination of Thiobarbituric Acid Reaction Substances (TBARSs)

Spanish mackerel samples (5 g) were weighed and placed in stoppered containers. Next, 50 mL of 7.5% trichloroacetic acid (in 0.1% ethylenediaminetetraacetic acid) was added to the samples. After oscillation and complete mixing, the samples were centrifuged at 5000 rpm for 10 min. TBARS analysis was performed as previously described [21].

### 2.10. Optimization of the Fermentation Conditions of FDSSM

Frozen Spanish mackerel was thawed in running water for 1.5 h. The head, tail, internal organs, and vertebrae were removed, and the fish were rinsed with running water. The fish were then cut into 5 cm × 5 cm cubes, with each cube weighing approximately 50–60 g. The excess surface water was then removed. Dry pickling was then performed by adding 4% (*w*/*w*) salt to pickle at 10 °C for 1 h, and then rinsing under running water to desalt. The effects of fermentation temperature (10 °C, 23.5 °C, or 37 °C), fermentation time (4 h, 6 h, or 8 h), and amount of bacteria added (2%, 4%, or 6%) on the quality of FDSSM samples were investigated using organoleptic evaluation as the criteria. Based on the results of the one-way experiments, the fermentation time, fermentation temperature, and amount of bacteria added were further optimized via response surface analysis according to the central combination experimental design principle of the Box–Behnken model.

### 2.11. Preparation of a Directed Vat-Set Starter for FDSSM

The preparation process for the directed vat-set starter involves the activation culture of the microbial strain, collection via centrifugation, addition of a freeze-drying stabilizer, freeze-drying, and the final product. Following preliminary experiments, the optimal conditions for minimizing bacterial cell loss and achieving the highest centrifugation yield (98.88 ± 0.22%) were determined with the following parameters: centrifugation speed of 3000 rpm, duration of 20 min, and temperature of 20 °C. A composite freeze-dried protective agent was prepared by blending 15% inulin, 5% Arabic gum, 5% sodium glutamate, and 5% skim milk powder.

Next, the fish meat was divided into three groups. Using the coating method, the fish samples were inoculated with a 4% (*v*/*v*) mixed microbial inoculum, as follows: DVSS samples, directed vat-set starter and LS+PP+LC samples, and mixed microbial solution containing LS, PP, and LC at a 1:1:1 ratio. The sample control underwent natural fermentation, and no exogenous microorganisms were introduced. The fish samples were fermented at a constant temperature of 28.5 °C for 5.5 h. Cold air-drying was then performed for 24 h with a set temperature of 20 °C, a bypass volume of 0.8, and an air velocity of 2 m/s. Sample CA was a commercially available sample. DC indicates the Spanish mackerel has completed the salting process; FJ3 indicates the Spanish mackerel has undergone fermentation for 3 h; FJ5.5 indicates the Spanish mackerel has undergone fermentation for 5.5 h; GZ14 indicates the Spanish mackerel has undergone cold air-drying for 14 h; GZ24 indicates the Spanish mackerel has undergone cold air-drying for 24 h.

#### 2.11.1. Determination of Live Bacteria Count in the Directed Vat-Set Starter

Inoculate the directed vat-set starter into MRS broth at a 1% inoculum volume. Incubate at 37 °C for 15–18 h, then activate the culture 2–3 times. Take the above bacterial suspension, centrifuge at 4 °C and 3000 rpm for 10 min, remove the supernatant, and wash the cells 2–3 times with sterile physiological saline. Add an appropriate volume of sterile physiological saline to the cells for enumeration.

#### 2.11.2. Study on the Storage Performance of the Directed Vat-Set Starter

Sealed samples of equal volumes of the directed vat-set starter were stored at −80 °C, −20 °C, 4 °C, and 20 °C. Viable cell counts were monitored after 15, 30, 45, and 60 days of storage. After storage, the directed vat-set starter was rehydrated with a sterile physiological saline solution of an equal volume to the pre-freeze-dried state. After thorough mixing, the cultures were diluted to the appropriate concentration. A 100 μL aliquot was spread onto solid MRS medium for incubation and subsequent enumeration.

#### 2.11.3. Effect of the Directed Vat-Set Starter on the pH During FDSSM Processing

The pH of each 3.0 g sample of Spanish mackerel meat was determined using a digital pH meter after homogenization in 27 mL of distilled water.

#### 2.11.4. Effect of the Directed Vat-Set Starter on Enzyme Activity During FDSSM Processing

Enzyme activity assays were conducted on the extracted Spanish mackerel samples, where 1 represents the blank sample, and 2–20 correspond to alkaline phosphatase, esterase, esterase lipase, lipase, leucine arylamidase, valine arylamidase, cystine arylamidase, trypsin, chymotrypsin, acid phosphatase, naphthol-AS-BI-phosphohydrolase, α-galactosidase, β-galactosidase, β-glucuronidase, α-glucosidase, β-glucosidase, N-acetyl-glucosaminidase, α-mannosidase, and fucosidase, respectively. A point scale from 0–5 was sued to indicate color intensity: 0 points indicates no enzyme activity; 1–2 points indicate negative enzyme activity; 3–5 points indicate positive enzyme activity.

### 2.12. Statistical Analysis

Each experiment was performed at least thrice. The results were analyzed using SPSS statistical analysis software (version 16.0; SPSS Inc., Chicago, IL, USA). Table 1 and Table 2 present the data as the mean ± standard deviation. One-way ANOVA with Tukey’s post-hoc test was used to compare the means. Statistical significance was set at *p* < 0.05. The wet weight of each sample was used to normalize the data.

## 3. Results and Discussion

### 3.1. Development of FDSSM Using a Composite Fermentation Agent and Analysis of Its Flavor Compounds

#### 3.1.1. Analysis of the Antagonistic Properties Among Different Bacterial Strains

**Figure 1:**
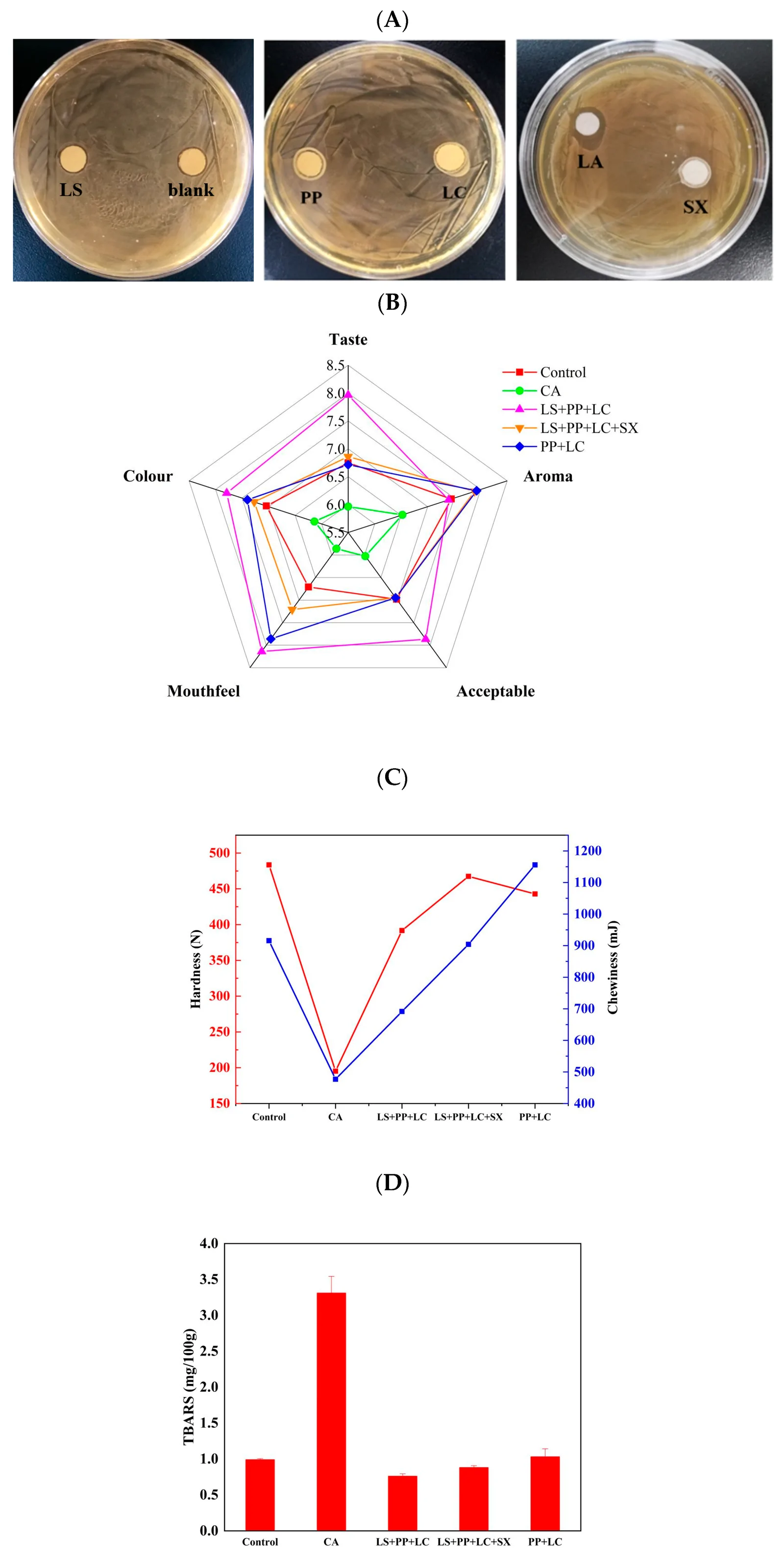
Results of antibiosis between different strains, sensory evaluation, texture and TBARS values for different samples. (**A**) Results of antibiosis between different strains. (**B**) Sensory evaluation results of fermented dried salted Spanish mackerel (FDSSM). (**C**) Texture profile analysis of fermented dried salted Spanish mackerel (FDSSM). (**D**) TBARS values for different fermented dried salted Spanish mackerel (FDSSM) samples. **Sample Control:** Natural fermentation (no exogenous microorganisms introduced). **Sample CA** represents a commercially available sample. **Sample LS+PP+LC**: Inoculated with a mixed microbial solution of LS+PP+LC in a 1:1:1 ratio. **Sample LS+PP+LC+SX**: Inoculated with a mixed microbial solution of LS+PP+LC+SX in a 2:2:2:1 ratio. **Sample PP+LC:** Inoculated with a mixed microbial solution of PP+LC in a 1:1 ratio.

Many lactic acid bacteria can produce bacteriocins that inhibit the growth of certain microorganisms. This mutual inhibitory effect can also occur among lactic acid bacteria, potentially leading to less effective fermentation with mixed cultures than with single strains [17]. This suggests that the combined fermentation cultures may not exhibit antagonistic interactions. As shown in Figure 1A, in plates inoculated with LS indicator bacteria, a distinct clear zone of inhibition only appeared around the filter paper discs containing the LA bacterial solution. Although extremely narrow clear zones were also observed around the control group and filter paper discs inoculated with other bacterial suspensions, comparisons indicated that these clear zones resulted from acid production. Furthermore, the figures clearly demonstrate that LS can coexist and grow with PP, LC, and SX. However, LA and LS exhibit significant antagonistic effects and are unsuitable for mixed fermentation. Conversely, LS, PP, LC, and SX can be co-cultured to prepare a mixed fermentation starter.

#### 3.1.2. Sensory Analysis of FDSSM Samples

Sensory evaluations were conducted for five samples. Figure 1B shows that the LS+PP+LC group demonstrated the highest acceptability across color, aroma, taste, and mouthfeel. It possesses the characteristic fresh aroma and rich flavor of salted fish, with a well-balanced saltiness, ample umami, complex taste, and a pleasant, chewy texture. Sample CA, which represents commercially available salted fish products, scored lower than the other four samples across all metrics. Its flesh exhibited poor coloration with noticeable browning, a faint fishy odor, a rancid taste, a bland flavor, and a lack of elasticity. Conversely, samples LS+PP+LC+SX, PP+LC, and the Control possessed a slightly fragrant aroma and were well-received by the evaluators, with their scores being relatively close.

#### 3.1.3. Flavor Nucleotide Analysis of FDSSM Samples

In fish, 5′-nucleotides are important taste-active molecules that contribute significantly to their umami flavor. AMP is the major umami 5′-nucleotide in seafood, including oysters and squid. IMP may help create a pleasant flavor profile such as meaty or brothy. As shown in Table 1, three 5′-nucleotides (AMP, CMP, and IMP) were detected in the five types of FDSSM samples. CMP was detected only in samples LS+PP+LC and LS+PP+LC+SX, with concentrations of 5.37 and 0.85 mg/100 g, respectively. Among the five samples, sample LS+PP+LC had the highest IMP and HxR content at 492.07 mg/100 g and 526.36 mg/100 g, respectively. Moreover, its levels of IMP, the main flavor-presenting nucleotides of FDSSM, were greater than the threshold of 25 mg/100 g. IMP produces synergistic umami effects with glutamic acid. The IMP content of the LS+PP+LC sample was significantly higher than that of the control. AMP and Hx were detected only in the LS+PP+LC samples, where the levels of AMP and Hx in fermented fish are mostly determined by the fish itself; however, different fermentation agents may also influence these levels. Collectively, the results of the nucleotide content analysis indicated that samples in the LS+PP+LC group exhibited the optimal nucleotide profile, suggesting that the LS+PP+LC products may possess superior taste characteristics.

#### 3.1.4. FAA Analysis of FDSSM Samples

The FAAs released during fermentation can increase the flavor and nutritional value of fermented fish [11]. FAAs generated through proteolysis contribute to flavor and odor generation in fermented fish, as they can be converted into volatile compounds via the transamination and Hofmann elimination pathways using microbial enzymes [22]. Table 2 lists the 17 FAAs identified in the FDSSM samples. Asp and Glu act as monosodium glutamate-like chemicals, giving FDSSM an umami flavor, whereas Gly, Ser, Ala, Thr, Pro, and Arg add a pleasant, sweet taste. In contrast, Tyr, Lys, Met, Phe, Val, Ile, Leu, and His impart bitter tastes. Glu is thought to be an important umami-enhancing component in fish. The Glu content in samples LS+PP+LC and PP+LC increased by 139.74% and 144.02%, respectively, compared to that of the control. The significant increase in Glu content in the fermented LS+PP+LC and PP+LC samples revealed enhanced freshness.

Furthermore, the LS+PP+LC and PP+LC groups had higher levels of the sweet amino acids Thr, Ala, and Pro than those in the Control group. Gly levels were significantly decreased in the LS+PP+LC, LS+PP+LC+SX, and PP+LC groups. In contrast, the Thr, Ala, and Pro concentrations were significantly higher than those in the Control group (*p* < 0.05). The total concentration of sweet amino acids increased from 0.961 to 1.194 mg/100 g, whereas the total FAA concentration in the FDSSM increased from 9.081 to 9.946 mg/100 g. The flavor and nutritional value of FDSSM improved significantly as the levels of pleasant-tasting amino acids increased from 5.381 to 6.779 mg/100 g (*p* < 0.05). These findings agree with those of Liu et al. (2021) [23] and Yang et al. (2020) [24]. Fermentation and maturation processes produce FAAs in beef products, whereas microbes aid in muscle protein degradation [25]. Hence, we postulated that during FDSSM fermentation, bacteria break down macromolecular proteins into smaller peptides and FAAs [26], thereby improving the nutritional value and flavor of FDSSM.

#### 3.1.5. TPA

The taste of fish is determined by its hardness and chewiness. TPA was used to investigate the textural changes between the different FDSSM samples. Figure 1C shows the variations in the textural attributes of the FDSSM samples. The results showed that fermentation considerably reduced the hardness of the FDSSM samples compared to the control (*p* < 0.05). For the LS+PP+LC and LS+PP+LC+SX samples, the hardness values dropped from 483.4 N to 391.7 N and 467.4 N, respectively. Similarly, the chewiness values decreased from 915.67 mj to 691.45 mj and 904.25 mj, respectively. However, after fermentation, the chewiness of the PP+LC samples increased to 1155.89 mj. This could be due to variances in fermentation strains, which influence the textural quality of the fermented fish.

Zhou et al. (2021) [27] discovered that the hardness, cohesiveness, and chewiness of stinky mandarin fish decreased initially and then increased with increased processing time during spontaneous fermentation at 8 °C. In our experiment, the hardness and chewiness of Spanish mackerel decreased after fermentation, which may be due to microbial fermentation, causing the degradation of fish protein and reducing the hardness and chewiness of fish meat.

#### 3.1.6. TBARS Analysis

During the curing and fermentation processes, body fat, and other compounds in fermented fish undergo complex chemical changes caused by microorganisms or external variables, such as temperature, humidity, and oxygen. Fat metabolism involves two pathways: fat hydrolysis and oxidation. Figure 1D depicts the variations in the TBARS values of Spanish mackerel throughout processing. The TBARS value of the five FDSSM groups ranged from 0.76 to 3.31 mg/100 g. The CA group exhibited the highest TBARS value, reflecting the degree of fat oxidation. Combined with the sensory evaluation results indicating rancid and oxidized flavors in the CA sample, excessive fat oxidation causes undesirable flavors in salt-dried fish. In contrast, the Control, LS+PP+LC, LS+PP+LC+SX, and PP+LC samples all showed significantly lower TBARS values than the CA sample, with LS+PP+LC and LS+PP+LC+SX exhibiting lower values than the PP+LC and Control samples. This may be attributed to LS, which decomposes proteins to generate antioxidant peptides and consequently inhibit lipid oxidation.

#### 3.1.7. HS-SPME-GC-MS Analysis and Analysis of the Characteristic Flavor Substances and Differential Volatiles of FDSSM Samples

The HS-SPME-GC-MS method was used to investigate the qualitative and quantitative changes in volatile taste chemicals in FDSSM during fermentation. The analysis revealed 77 volatile compounds across five samples, including 16 aldehydes, 11 alcohols, 6 ketones, 10 esters, 17 alkanes, and 17 other compounds (Table 3). Samples LS+PP+LC, LS+PP+LC+SX, PP+LC, Control, and CA were found to contain 41, 37, 35, 32, and 51 major volatile flavor compounds, respectively, with total relative contents of 68.53%, 64.89%, 71.95%, 66.12%, and 66.84% (Table 4), respectively. Samples LS+PP+LC, LS+PP+LC+SX, PP+LC, and the Control, which were processed using identical techniques, exhibited certain similarities in their volatile flavor compound profiles. Hydrocarbons had the highest relative content; aldehydes, alcohols, and heterocyclic compounds were also relatively abundant. However, differences existed among these four samples: sample LS+PP+LC exhibited a significantly higher relative ester content than the others, while the PP+LC and Control samples contained markedly higher levels of aldehydes and ketones than LS+PP+LC and LS+PP+LC+SX samples. CA had the highest relative aldehyde content, followed by ketones, hydrocarbons, alcohols, and heterocyclic compounds. These results indicate that aldehydes, alcohols, hydrocarbons, and heterocyclic compounds constitute the primary volatile flavor components in FDSSM samples. Differences in processing techniques and environments may lead to significant variations in the volatile flavor compounds of FDSSM. Furthermore, samples produced using identical processing methods exhibited distinct flavor profiles owing to variations in the fermentation agents employed.

The relative contents of esters and heterocyclic compounds in the LS+PP+LC, LS+PP+LC+SX, and PP+LC samples were higher than those in the natural fermentation groups (Control and CA samples). The difference in ester content was particularly significant, especially in sample LS+PP+LC, where the relative ester content reached 15.27%, significantly higher than the 0.80% and 0.53% in the Control and CA samples, respectively. This was attributed to the introduction of exogenous microorganisms that increased the production of acids and alcohols via microbial metabolism. These compounds undergo esterification reactions, leading to a higher ester content [28,29]. Heterocyclic compounds have a lower threshold, with pyrazines and furans contributing to mild aromas and nutty notes. Thus, the presence of these compounds promoted the formation of favorable flavors in the FDSSM samples [30].

The relative contents of aldehyde and ketone compounds in the experimental groups supplemented with exogenous microorganisms (LS+PP+LC, LS+PP+LC+SX, and PP+LC samples) were almost uniformly lower than those of the naturally fermented control group (Control and CA samples). This is because aldehydes and ketones possess relatively more reactive chemical properties and act as unstable intermediates that readily undergo further reduction to the corresponding acids or alcohols under the influence of exogenous microorganisms. In contrast, the fermentation process in naturally fermented samples was initiated more slowly, resulting in the reduction in fewer aldehydes and ketones. Consequently, the levels of residual aldehydes and ketones in these samples were higher than those in the artificially inoculated fermentation products [30]. Aldehydes possess a low sensory threshold and contribute significantly to the flavor profile of FDSSM [31]. In contrast, ketones can be generated through amino acid degradation, the oxidation of PUFAs, or microbial oxidation [32,33]. Although their sensory thresholds are relatively high and their contribution to flavor is comparatively minor, the presence of ketones can intensify or alter their fishy odors.

The alcohols and hydrocarbons in the five sample groups exhibited no significant differences or discernible patterns. The proportion of alcohols in each sample remained at approximately 7.00%. These alcohols primarily originated from the oxidative decomposition of fats and the reduction of sugars, amino acids, aldehydes, and other compounds. Through natural or artificial fermentation, the alcohol content increases continuously while complex biochemical reactions happen, such as aldehyde reduction and lipid oxidation. However, the esterification and oxidation reactions simultaneously caused a gradual decrease. Hydrocarbons are primarily formed through the homolytic cleavage of fatty acid alkoxy radicals, but their threshold is relatively high, generally contributing little to food flavor [34].

Food contains a wide range of flavor compounds with varied sensory contributions; therefore, it is critical to identify the main components responsible for these distinct flavors. The taste threshold is the lowest concentration that is noticeable in humans. The relative odor activity value (ROAV), which assesses the contribution of a compound to the overall flavor, was calculated using the relationship between its concentration and the threshold. Generally, substances with ROAVs > 1 are recognized as characteristic flavor compounds, with substances with ROAVs between 0.1 and 1 also exerting a relatively significant influence on flavor [35]. Due to its relatively high content ranging from 3.02% to 12.51% and low sensory threshold of 0.2 μg/kg, 3-methylbutanal contributes most significantly to the flavor profile of FDSSM. Therefore, the ROAV of 3-methylbutanal was defined as ROAVstan = 100. The ROAVs for other volatile flavor components were calculated using the formula presented in Table 5.

Based on the volatile compound concentrations and their corresponding threshold values (Table 5), the key flavor compounds in FDSSM primarily include 3-methylbutanal, *n*-hexanal, *n*-heptanal, *n*-octanal, nonanal, 1-octen-3-ol, and heptanol. Aldehydes are primarily produced by the cracking of unsaturated fatty acids and contribute significantly to flavor [23,31]. Hexanal, heptanal, nonanal, octanal, and benzaldehyde were detected in all samples, whereas 3-methylbutanal was present at high concentrations in all samples except LS+PP+LC. These six major aldehydes constitute the primary contributors to the flavor of salted fish.

Lu et al. (2010) [36] indicated that the key flavor compounds in yellow croaker, redfish, and sea bass are saturated straight-chain aldehydes, such as hexanal, heptanal, and nonanal. Alcohol compounds are primarily generated through various reactions, such as carbohydrate decomposition, fat oxidation, and amino acid reduction. Volatile alcohols produce a delicate aroma, imparting a mild and fragrant flavor to fermented fish meat [4,37]. Table 5 indicates that 1-octen-3-ol, cis-2-penten-1-ol, and heptanol are the primary flavor compounds in FDSSM; they are present in all five samples. Ketone compounds exhibit high detection thresholds and contribute minimally to food flavor at low concentrations [33].

Esters are excellent flavor compounds, with small amounts producing distinct aromas [24,38]. Short-chain esters possess extremely low detection thresholds (10^−9^ level), making them easily perceivable to individuals who are highly sensitive to taste perception. They impart a characteristic aroma to fermented products and contribute significantly to overall flavor intensity. Samples of esters include the following: benzyl acetate, a commonly used flavor ingredient with a distinctive jasmine-like aroma, and methyl o-aminobenzoate, a food flavoring that possesses a grape-like scent. Therefore, whether evaluated via relative content or flavor contribution, esters are the primary flavor compounds unique to LS+PP+LC samples.

The results shown in Table 5 indicate that LS+PP+LC samples exhibit significantly higher ROAVs for saturated straight-chain aldehydes such as hexanal, heptanal, and nonanal, as well as several major alcohols, compared to other samples. This suggests that the superior flavor quality of LS+PP+LC samples is likely attributable to the dominant contribution of key flavor-enhancing compounds.

Comparing LS+PP+LC and PP+LC samples, the results suggest that LS—an endogenous dominant strain traditionally introduced in salted dried Spanish mackerel—may have been uniquely incorporated into LS+PP+LC samples. As the primary microorganism in second-generation fermentation agents, *Lactobacillus* strains exhibit strong competitiveness, capable of suppressing the growth of certain lactic acid bacteria in natural environments and dominating the entire fermentation and drying process [2]. Meanwhile, LS can also naturally proliferate in salted dried Spanish mackerel and affect the fermentation process as the predominant bacterial strain, imparting its distinctive flavor and texture. The exogenous introduction of LS further increases its concentration, enhancing fermentation efficiency. This likely explains why sample LS+PP+LC exhibited superior aroma characteristics and flavor profiles compared to other samples.

Comparison of the test results of samples LS+PP+LC and LS+PP+LC+SX revealed that SX confers a unique flavor profile to LS+PP+LC+SX samples; however, it does not confer any beneficial effects. Meanwhile, staphylococci can theoretically impart distinctive flavors to fermented products and play a crucial role in maintaining color and aroma stability [14]. In previous studies, GC-IMS analysis was performed to investigate the difference between the flavor compounds of fermented stinky sea bass. The results show that 46 volatile flavor compounds were identified, including 14 alcohols, 7 esters, 9 ketones, 6 aldehydes, and other compounds such as acids. Acetic acid and acetoin were detected in all eight sea bass meat samples. The acetic acid and acetoin content in the FSSBs increased as the fermentation proceeded [7]. Fifty-nine volatile compounds (including monomers and dimers), such as alcohols, aldehydes, esters, ketones, and acids, were detected in the wine lees golden pomfret samples from the three processing stages, XY, GZ, and FJ. Acetone, acetic acid, and ethyl propionate were detected at all three stages of processing. 2-Methylbutyraldehyde, which imparts a hazelnut and malt aroma; 2,3-dibutanone, which imparts a butter flavor; and methyl acetate, which has a specific ester aroma, were only detected in the XY samples and may be the key flavor substances in fresh golden pomfret [9].

Microbial fermentation is a complex process. During mixed fermentation, interactions among different bacterial strains may prevent the beneficial effects of individual strains from being fully realized. CA undergoes fermentation under natural conditions involving numerous uncertainties and an extended production period. Microbes of various species and quantities that accumulated in the fish meat, along with the transformation of flavor compounds, differed significantly from those in the other four samples. This results in the presence of multiple unique flavor compounds, such as trans-2-octenal and trans-2-nonenal. Analysis of the ROAVs indicated that some compounds, primarily (Z)-4-heptenal and trans-2-nonenal, significantly contributed to the flavor profile of CA. This explains the pronounced differences in flavor characteristics between CA and the other samples.

### 3.2. Optimization of the Fermentation Conditions of FDSSM

**Figure 2:**
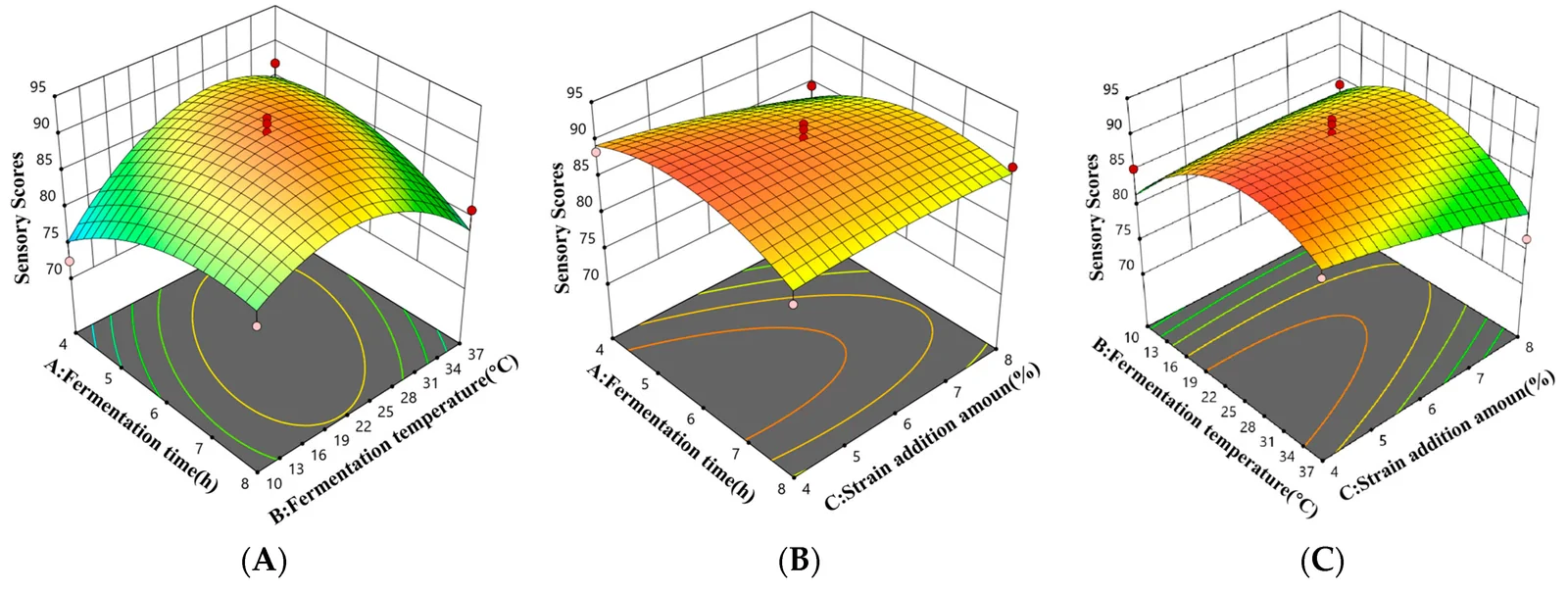
The effect of interaction among various factors on the sensory evaluation of fermented dried salted Spanish mackerel (FDSSM). (**A**) The effect of interaction among fermentation time and fermentation temperature on the sensory evaluation of fermented dried salted Spanish mackerel (FDSSM). (**B**) The effect of interaction among fermentation time and strain addition amount on the sensory evaluation of fermented dried salted Spanish mackerel (FDSSM). (**C**) The effect of interaction among fermentation temperature and strain addition amount on the sensory evaluation of fermented dried salted Spanish mackerel (FDSSM).

Based on the findings of the single-factor trials, response surface methodology was utilized to improve the processing process for three factors: fermentation temperature, fermentation time, and amount of bacteria added. Multiple fitting regression analysis of the Box–Behnken test data resulted in the following regression model equation for the FDSSM fermentation conditions, Y = 90.68 + 0.49A + 1.03B − 1.69C − 3.83AB + 1.55AC − 2.42BC − 3.44A^2^ − 6.57B^2^ − 0.64C^2^, with Y denoting the sensory score (Figure 2A–C, Supplementary Tables S1 and S2). This regression model showed a highly significant difference (*p* < 0.0001), whereas the misfit term was not significant. The model has a coefficient of determination R^2^ = 82.69% and a correction coefficient R^2^adj = 60.42%, showing good fit and that the test results are very close to the anticipated values [39]. From the F-values, the order of the factors in affecting the quality of the FDSSM preparation was B (fermentation temperature) > A (fermentation time) > C (strain addition amount).

Under the optimum fermentation time of 5.28 h, fermentation temperature of 28.45 °C, and strain addition amount of 4%, the sensory score of the FDSSM samples reached 92.55 points. The FDSSM processing parameters were then adjusted as follows to match the actual production conditions: fermentation time, 5.5 h; fermentation temperature, 28.5 °C; and strain addition amount, LS+PP+LC 4%. These settings served as the basis for the validation tests. The sensory score of the FDSSM samples when utilizing these parameters was 91.5, which is close to the predicted value, indicating that the process parameters produced from the model were accurate.

The final production process for the FDSSM is as follows. Frozen Spanish mackerel was thawed in running water for 1.5 h. The head, tail, internal organs, and vertebrae were then removed, and the fish were rinsed with running water. Next, the fish were cut into 5 cm × 5 cm cubes, with each cube weighing approximately 50–60 g. The excess surface water was then removed. Dry pickling was then performed, adding 4% (*w*/*w*) salt to pickle at 10 °C for 1 h, and then rinsing under running water to desalt. The fermentation conditions for FDSSM were as follows: fermentation time of 5.5 h at 28.5 °C, with a strain addition amount of LS+PP+LC at 4%. Cold air-drying was then performed for 24 h with a set temperature of 20 °C, a bypass volume of 0.8, and an air velocity of 2 m/s.

### 3.3. Preparation of a Directed Vat-Set Starter of FDSSM

A directed vat-set starter is a high-potency, high-concentration, standardized solid fermentation agent produced by drying lactic acid bacteria and packaging them under sterile conditions [16]. Compared to liquid bacterial starter cultures, directed vat-set starters offer advantages such as ease of use, transportation, and storage, as well as high fermentation potency [3]. In practical applications, they effectively reduce production costs, shorten production cycles, and enhance product stability and safety [40]. Therefore, the development of a directed vat-set starter suitable for FDSSM processing is of utmost importance.

#### 3.3.1. Research on Composite Freeze-Dried Preservatives for FDSSM

Multiple protective agents with distinct mechanisms are often combined to enhance the survival rate of microbial cells upon freeze-drying. The pre-experimental findings demonstrated that the bacterial strains were not well preserved by the sole cryoprotectant (Supplementary Table S3). In this study, a synergistic approach leveraged the cumulative effects of multiple protective actions to improve strain survival during freeze-drying. Herein, a preservative mixture was created containing 5% skim milk powder, 5% monosodium glutamate, 15% inulin, and 5% gum arabic in different combinations to investigate the protective effects of different component combinations on freeze-drying. Table 6 presents the results. All combinations demonstrated superior freeze-drying protection compared with the individual agents, with varying degrees of improvement in strain survival rates. The composite protective agent containing 15% inulin, 5% gum arabic, 5% skim milk powder, and 5% sodium glutamate at a 1:1:1:5 ratio achieved the highest survival rate upon freeze-drying, at 69.86 ± 0.23%. At this point, the viable bacterial count in the direct vat-set starter was (3.19 ± 0.02) × 10^9^ CFU/g.

#### 3.3.2. Study on the Storage Stability of the Directed Vat-Set Starter

**Figure 3:**
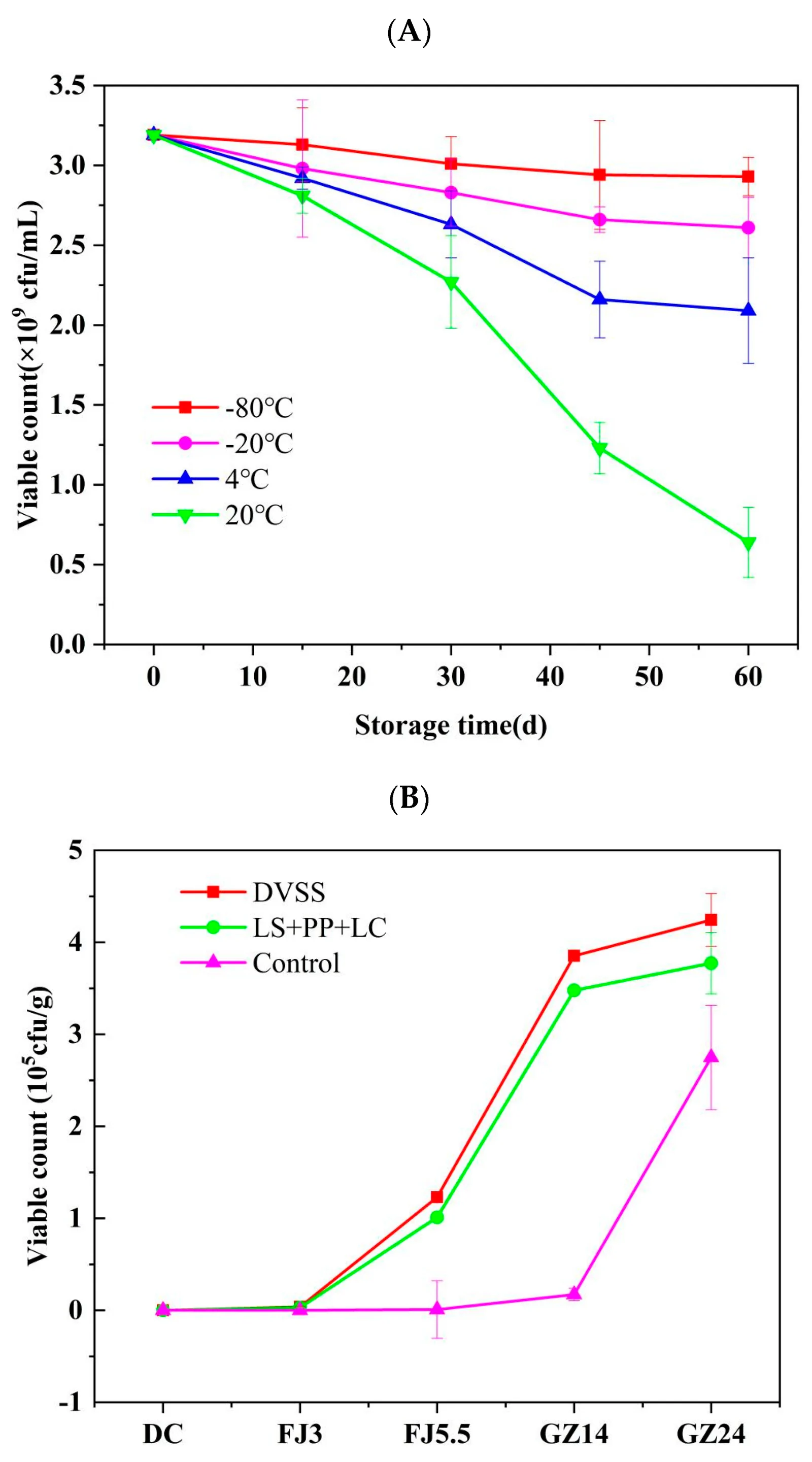

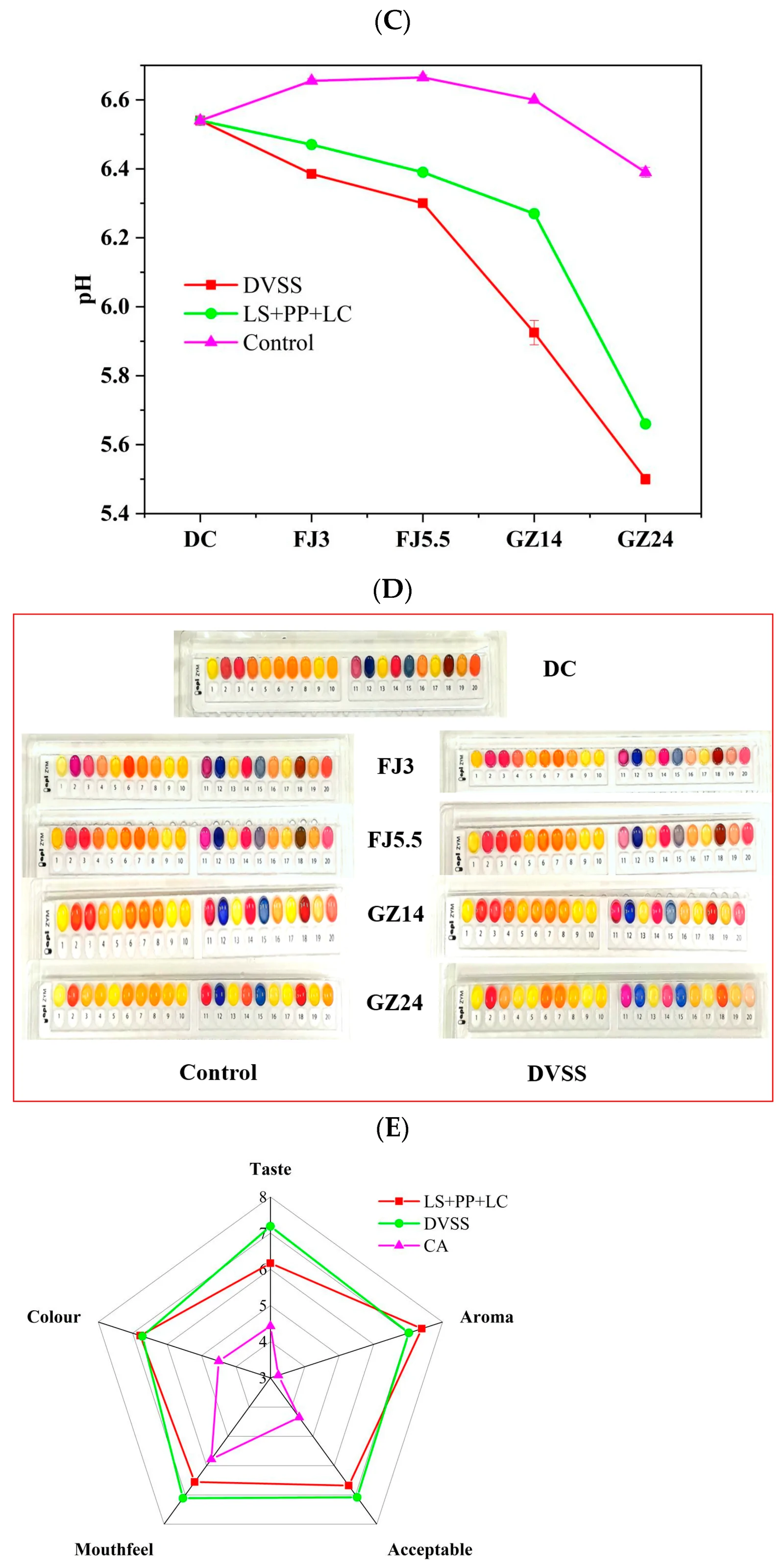
**Preparation of directed vat-set starter of fermented dried salted Spanish mackerel (FDSSM).** (**A**) Study on the storage stability of directed vat-set starter. (**B**) Study on the fermentation activity of directed vat-set starter during FSDDM processing. (**C**) Determination of pH during FSDDM processing. (**D**) Determination of enzyme activity during FSDDM processing. (**E**) The sensory evaluation of DVSS, LS+PP+LC and CA samples. **Sample Control:** Natural fermentation (no exogenous microorganisms introduced). **Sample CA** represents a commercially available sample. **Sample DVSS:** Inoculated with directed vat-set starter. **Sample LS+PP+LC:** Inoculated with a mixed microbial solution of LS+PP+LC in a 1:1:1 ratio. **DC**: The Spanish mackerel has completed the salting process. **FJ3**: The Spanish mackerel has undergone fermentation for 3 h. **FJ5.5**: The Spanish mackerel has undergone fermentation for 5.5 h. **GZ14**: The Spanish mackerel has undergone cold air drying for 14 h. **GZ24** indicates the Spanish mackerel has undergone cold air drying for 24 h.

Storage stability and fermentation activity are key indicators of the quality of a directed vat-set starter [3]. Moreover, temperature is a critical factor that determines storage stability, and different storage temperatures can significantly affect the fermentation activity of microbial cultures [16]. As shown in Figure 3A, after 60 days of storage at −80 °C, the viable bacterial count decreased from the initial (3.19 ± 0.02) × 10^9^ CFU/g to (2.93 ± 0.12) × 10^9^ CFU/g, representing a decline of 8.15%. After 60 days of storage at −20 °C, the viable bacterial count decreased by 18.19%. After 60 days of storage at 4 °C, the viable bacterial count dropped to (2.09 ± 0.33) × 10^9^ CFU/g, a decrease of 34.48%. Finally, after 60 days of storage at 20 °C, the live bacteria count in the starter culture decreased the most, reaching 79.94%. However, under all temperature conditions, the live bacteria count in the starter culture remained above 10^9^ CFU/g, demonstrating excellent storage performance.

At different storage temperatures, the viable cell counts for each fermentation agent exhibited a declining trend over time, with the rate of decline being directly proportional to the storage temperature, as higher temperatures resulted in greater and faster reductions in viable cell counts. This indicates that lower storage temperatures yield higher microbial survival rates, thereby extending the effective shelf life of fermentation agents.

#### 3.3.3. Study on the Fermentation Activity of Directed Vat-Set Starter During FSDDM Processing

As shown in Figure 3B, the viable bacterial counts in all three sample groups increased throughout the process. However, compared to the Control group, the experimental groups DVSS and LS+PP+LC with added fermentation agents achieved substantial proliferation in a shorter timeframe, exhibited faster growth rates, and had higher viable bacterial counts in the final products than the other groups. This is because the raw material for frozen Spanish mackerel inherently contains almost no viable bacteria, making natural fermentation ineffective for achieving the desired results within a short timeframe. Consequently, the viable bacterial count remained virtually unchanged during the early stages of processing and only began to increase rapidly during the later stages of drying. Additionally, the trend in viable bacterial counts in the DVSS and LS+PP+LC samples was nearly identical, with both ultimately reaching approximately 4.0 × 10^5^ CFU/g. However, the DVSS samples exhibited a faster growth rate and higher final viable bacterial count than the LS+PP+LC samples. This is because under the protective and promotive effects of the preservative, DVSS bacteria proliferate faster than other bacterial cultures, producing large amounts of lactic acid within a short period. This inhibited the growth of other bacterial species, allowing DVSS to become the dominant strain.

#### 3.3.4. Determination of pH During FSDDM Processing

The Control sample exhibited a pH trend that initially increased slightly before gradually declining, with the pH of the final product stabilizing at approximately 6.4 (Figure 3C). This occurred because during the early stages of natural fermentation, lactic acid bacteria were in a slow growth phase, producing minimal acid, and not yet becoming the dominant microbial strain in the fish meat environment. At this point, proteins are degraded by proteases to produce non-protein nitrogen [11], causing a slight increase in pH. As fermentation progresses, the proliferation rate of lactic acid bacteria increases, leading to the accumulation of lactic acid and a gradual decrease in pH. During FSDDM processing, the pH values of the DVSS and LS+PP+LC samples continuously decreased, and the rate of decline gradually increased. This is because the artificially inoculated microorganisms enabled the lactic acid bacteria to become the dominant strain during the early fermentation stage, allowing them to proliferate rapidly. They metabolize carbohydrates to produce acids at a high rate, maintaining a consistently declining pH. Although the pH values of DVSS and LS+PP+LC samples showed a decreasing trend, the extent of the decrease varied significantly. The pH of DVSS decreased more rapidly, and the final product also exhibited a lower pH value than the LS+PP+LC samples. This may be attributed to the presence of bacteria proliferating at a higher rate under the protection of preservatives and the action of growth promoters, leading to accelerated acid production. These results indicate that the directly inoculated lactic acid bacteria starter culture developed in this study possesses excellent acid-producing capabilities.

#### 3.3.5. Determination of Enzyme Activity During FDSSM Processing

Analysis of enzyme activity during FDSSM processing revealed that the primary enzymes involved in the processing of the Control sample include alkaline phosphatase, leucine arylamidase, valine arylamidase, acid phosphatase, naphtol-AS-BI-phosphohydrolase, β-galactosidase, and N-acetyl-glucosaminidase (Figure 3D). The primary enzymes involved in the processing of the DVSS sample include alkaline phosphatase, valine arylamidase, acid phosphatase, naphthol-AS-BI-phosphohydrolase, β-galactosidase, and N-acetyl-glucosaminidase. These differences in enzyme activity may be related to the quality and flavor of the FDSSM product.

#### 3.3.6. Sensory Analysis of FDSSM Fermented Using Directed Vat-Set Starter

Sensory evaluation indicated that the CA samples possessed a fragrant aroma, with an overall acceptability score of 6.68 (Figure 3E, Supplementary Figure S1). In contrast, the commercially available LS+PP+LC sample exhibited pale, dull flesh with a rancid odor and a pronounced fishy taste. The fish products were mushy and lacked elasticity and chewiness. The DVSS sample achieved the highest overall acceptability, with a composite score of 7.08. The fish meat in this sample was firm, elastic, and chewy, featuring a rich flavor, ample umami, and the characteristic savory depth and mellow aroma of salted fish. This superior profile resulted from the freeze-drying protectants in the bacterial powder containing growth-promoting factors for the strains, enabling heightened fermentation activity and accelerating the development of desirable flavors. Simultaneously, the presence of lactic acid bacteria lowered the pH of fish meat through acid production. When the pH approaches the isoelectric point (pI = 5.4) of muscle proteins, they form gel-like structures under acidic conditions. This increases the cohesion between meat fibers, enhancing the elasticity and fineness of fish meat while improving the overall texture. Therefore, the addition of directly inoculated fermentation agents resulted in the expected improvement in the sensory quality of FDSSM.

## 4. Conclusions

This study aimed to develop an FDSSM product using a composite fermentation agent and analyze its flavor fermentation, fermentation conditions, and the effect of preparing a directed vat-set starter. The results showed that the FDSSM products fermented with LS+PP+LC at a 1:1:1 ratio exhibited the best sensory quality. Moreover, LS+PP+LC samples exhibited the highest 5′-IMP and HX content at 492.07 and 526.36 mg/100 g, respectively. After fermentation with the composite starter culture, the concentration of umami amino acids in FDSSM significantly increased, while the TBARS levels significantly decreased. Hardness and chewiness were significantly higher than those of the CA sample. A total of 77 flavor compounds were detected across the five samples. The characteristic flavor substances of FDSSM were: 3-methylbutanal, *n*-hexanal, *n*-heptanal, *n*-octanal, nonanal, 1-octen-3-ol, and heptanol. The optimal fermentation conditions for FDSSM were a fermentation time of 5.5 h, a fermentation temperature of 28.5 °C, and a strain addition amount of 4%. Moreover, the preservative mixture containing 15% inulin, 5% Gum arabic, 5% skim milk powder, and 5% sodium glutamate in a 1:1:1:5 ratio demonstrated the highest microbial protection rate of 69.86 ± 0.23%. The viable bacterial count in the starter cultures stored for 60 days exceeded 10 CFU/g. Furthermore, the optimal storage temperature for the direct-inoculation starter cultures was −20 °C. Finally, the addition of a directed vat-set starter yielded superior sensory quality in FDSSM compared with mixed bacterial cultures. Future research should be conducted on the physicochemical properties, flavor formation, metabolomics, and microbial diversity of FDSSM.

## Figures and Tables

**Table 1:** Flavor nucleotide content (mg/100 g) and TAV values in 5 fermented dried salted Spanish mackerel (FDSSM) samples.

Flavor Nucleotide	Control	CA	LS+PP+LC	LS+PP+LC+SX	PP+LC
5′-CMP	ND	ND	5.37 ± 0.08	0.85 ± 0.03	ND
5′-IMP	204.32 ± 2.96 ^b^	286.40 ± 4.50 ^c^	492.07 ± 1.68 ^d^	281.31 ± 5.40 ^c^	93.19 ± 3.15 ^a^
Hx	ND	ND	17.43 ± 1.25	ND	ND
5′-AMP	ND	ND	5.42 ± 0.50	ND	ND
HxR	175.56 ± 0.99 ^c^	93.55 ± 0.63 ^b^	526.36 ± 6.32 ^d^	180.89 ± 12.74 ^c^	75.30 ± 2.31 ^a^
Total	379.88 ± 3.95 ^b^	379.94 ± 4.93 ^b^	1046.64 ± 6.60 ^d^	463.04 ± 15.34 ^c^	168.49 ± 5.42 ^a^
TVA (5′-AMP)	ND	ND	0.108 ± 0.01	ND	ND
TVA (5′-IMP)	8.173 ± 0.12 ^b^	11.456 ± 0.18 ^c^	19.683 ± 0.07 ^d^	11.252 ± 0.22 ^c^	3.728 ± 0.13 ^a^

**Sample Control:** Natural fermentation (no exogenous microorganisms introduced). **Sample CA** represents a commercially available sample. **Sample LS+PP+LC:** Inoculated with a mixed microbial solution of LS+PP+LC in a 1:1:1 ratio. **Sample LS+PP+LC+SX:** Inoculated with a mixed microbial solution of LS+PP+LC+SX in a 2:2:2:1 ratio. **Sample PP+LC:** Inoculated with a mixed microbial solution of PP+LC in a 1:1 ratio. Author2026-8-14 20:36 GMT+0700Values are presented as mean ± SD (n = 3). Within the same row, different superscript letters (a–d) denote statistically significant differences among groups (*p* < 0.05);ND indicates no detection.

**Table 2:** Contents of free amino acids for different fermented dried salted Spanish mackerel (FDSSM) samples.

Free Amino Acids	Mouthfeel	Control	CA	LS+PP+LC	LS+PP+LC+SX	PP+LC
Asp	umami	5.186 ± 0.088 ^b^	10.133 ± 0.243 ^d^	4.651 ± 0.081 ^a^	5.664 ± 0.275 ^c^	4.429 ± 0.174 ^a^
Glu	umami	0.234 ± 0.003 ^a^	0.271 ± 0.012 ^b^	0.327 ± 0.002 ^c^	0.223 ± 0.002 ^a^	0.337 ± 0.005 ^c^
Thr	sweet	0.182 ± 0.028 ^a^	0.585 ± 0.011 ^c^	0.242 ± 0.013 ^b^	0.183 ± 0.039 ^a^	0.229 ± 0.031 ^b^
Ser	sweet	0.117 ± 0.036 ^bc^	0.003 ± 0.002 ^a^	0.079 ± 0.060 ^b^	0.043 ± 0.031 ^ab^	0.162 ± 0.023 ^c^
Gly	sweet	0.181 ± 0.001 ^d^	0.267 ± 0.003 ^e^	0.126 ± 0.000 ^a^	0.159 ± 0.000 ^b^	0.166 ± 0.001 ^c^
Ala	sweet	0.423 ± 0.001 ^a^	0.686 ± 0.004 ^e^	0.457 ± 0.001 ^c^	0.443 ± 0.002 ^b^	0.542 ± 0.000 ^d^
Pro	sweet	0.058 ± 0.001 ^a^	0.164 ± 0.001 ^e^	0.068 ± 0.000 ^c^	0.064 ± 0.001 ^b^	0.095 ± 0.001 ^d^
Val	bitter	0.116 ± 0.004 ^a^	0.321 ± 0.002 ^e^	0.162 ± 0.000 ^c^	0.141 ± 0.001 ^b^	0.208 ± 0.002 ^d^
Met	bitter	0.075 ± 0.007 ^a^	0.113 ± 0.000 ^c^	0.100 ± 0.001 ^b^	0.073 ± 0.000 ^a^	0.118 ± 0.009 ^c^
Ile	bitter	0.059 ± 0.002 ^a^	0.211 ± 0.004 ^e^	0.103 ± 0.000 ^c^	0.076 ± 0.000 ^b^	0.125 ± 0.005 ^d^
Leu	bitter	0.085 ± 0.001 ^a^	0.390 ± 0.002 ^e^	0.159 ± 0.000 ^c^	0.128 ± 0.000 ^b^	0.208 ± 0.002 ^d^
Tyr	bitter	0.073 ± 0.001 ^a^	0.173 ± 0.004 ^e^	0.108 ± 0.001 ^c^	0.091 ± 0.002 ^b^	0.121 ± 0.002 ^d^
Phe	bitter	0.059 ± 0.000 ^a^	0.222 ± 0.003 ^e^	0.091 ± 0.001 ^b^	0.087 ± 0.001 ^c^	0.115 ± 0.001 ^d^
Lys	bitter	0.531 ± 0.000 ^d^	0.451 ± 0.001 ^c^	0.403 ± 0.004 ^b^	0.290 ± 0.007 ^a^	0.620 ± 0.000 ^e^
His	bitter	1.606 ± 0.002 ^b^	1.626 ± 0.015 ^b^	1.785 ± 0.012 ^c^	1.455 ± 0.002 ^a^	2.311 ± 0.003 ^d^
Arg	bitter	0.096 ± 0.001 ^c^	0.050 ± 0.001 ^a^	0.158 ± 0.001 ^d^	0.091 ± 0.002 ^b^	0.158 ± 0.001 ^d^
Cys	/	0.000 ± 0.000 ^a^	0.026 ± 0.000 ^b^	0.000 ± 0.000 ^a^	0.000 ± 0.000 ^a^	0.000 ± 0.000 ^a^
Umami Amino Acids		5.420 ± 0.179 ^b^	10.404 ± 0.254 ^d^	4.978 ± 0.081 ^a^	5.887 ± 0.274 ^c^	4.766 ± 0.179 ^a^
Sweet Amino Acids		0.961 ± 0.060 ^ab^	1.706 ± 0.021 ^d^	0.972 ± 0.046 ^b^	0.892 ± 0.011 ^a^	1.194 ± 0.008 ^c^
Bitter Amino Acids		2.700 ± 0.016 ^b^	3.556 ± 0.030 ^d^	3.069 ± 0.017 ^c^	2.432 ± 0.006 ^a^	3.985 ± 0.024 ^e^
total pleasant-tasting amino acid		5.381 ± 0.013 ^a^	12.110 ± 0.275 ^d^	5.950 ± 0.039 ^b^	6.779 ± 0.284 ^c^	5.960 ± 0.187 ^b^
Total Amino Acids		9.081 ± 0.119 ^a^	15.691 ± 0.305 ^c^	9.048 ± 0.032 ^a^	9.211 ± 0.289 ^a^	9.946 ± 0.211 ^b^

**Sample Control:** Natural fermentation (no exogenous microorganisms introduced). **Sample CA** represents a commercially available sample. **Sample LS+PP+LC:** Inoculated with a mixed microbial solution of LS+PP+LC in a 1:1:1 ratio. **Sample LS+PP+LC+SX:** Inoculated with a mixed microbial solution of LS+PP+LC+SX in a 2:2:2:1 ratio. **Sample PP+LC:** Inoculated with a mixed microbial solution of PP+LC in a 1:1 ratio. Values are presented as mean ± SD (n = 3). Within the same row, different superscript letters (a–e) denote statistically significant differences among groups (*p* < 0.05).

**Table 3:** Volatile flavor compounds and their relative contents of five fermented dried salted Spanish mackerel (FDSSM) samples.

Category	Components	CAS	R.T. (min)	Relative Content (%)				
				Control	CA	LS+PP+LC	LS+PP+LC+SX	PP+LC
**Aldehydes**	Hexanal	66-25-1	7.42	4.48	6.04	3.41		2.83
	Heptanal	111-71-7	10.02	1.72	2.51	1.44	1.76	1.35
	Octanal	124-10-3	12.28	1.86	4.86	1.35	1.47	1.35
	Nonanal	124-19-6	14.28	1.28	3.27	1.19	1.32	0.87
	Benzaldehyde	100-52-7	16.56	2.53	1.43	1.91	1.17	1.24
	2,6-Nonadienal, (E,E)-	17587-33-6	17.52		0.80	0.30	0.23	
	Butanal, 3-methyl-	590-86-3	3.43	8.69	3.02		4.23	12.51
	Tetradecanal	124-25-4	24.89	0.40				
	2-Pentenal, (E)-	1576-87-0	7.71		0.90			
	2-Pentenal, 2-methyl-	623-36-9	9.35		0.38			
	2-Hexenal, (E)-	6728-26-3	10.76		1.17			
	4-Heptenal, (Z)-	6728-31-0	11.31		1.25			
	2-Octenal, (E)-	2548-87-0	14.91		0.34			
	2,4-Heptadienal, (E,E)-	4313-03-5	15.55		1.58			
	2-Nonenal, (E)-	18829-56-6	16.70		0.29			
	Benzaldehyde, 4-ethyl-	4748-78-1	19.39		0.28			
**Alcohols**	2-Penten-1-ol, (Z)-	1576-95-0	12.91	1.73	1.73	1.38	1.71	2.06
	3-Hexen-1-ol, (Z)-	928-96-1	14.12			0.27		
	1-Octen-3-ol	3391-86-4	15.24	2.19	2.86	1.14	1.85	1.95
	1-Heptanol	111-70-6	15.35	1.11	0.89	0.62	0.69	1.10
	1,6-Octadien-3-ol, 3,7-dimethyl-	126-90-9	16.84			0.45		
	Phenylethyl Alcohol	60-12-8	22.17		0.20	0.75		
	n-Heptadecanol-1	1454-85-9	27.62			0.36		
	2-Butanone, 3-hydroxy-						3.59	
	Benzyl Alcohol	100-51-6	21.70			1.81	0.26	
	1-Butanol, 3-methyl-	123-51-3	10.59	1.39				1.57
	3-Pentanol, 2-methyl-	565-67-3	13.35		0.34			
	1-Octanol	111-87-5	17.03		0.67			
**Ketones**	3,5-Octadien-2-one	30086-02-3	16.44		7.62	1.58	1.07	1.72
	3,5-Octadien-2-one, (E,E)-	30086-02-3	17.26	1.49	3.83	0.87	0.76	1.25
	2-Butanone, 3-hydroxy-	513-86-0	12.22	2.42				3.30
	2,3-Octanedione	585-25-1	13.02	1.68				1.05
	2-Nonanone	821-55-6	14.18	0.27	0.99			0.23
	2-Undecanone	112-12-9	17.67		0.52			
**Esters**	Butanoic acid, butyl ester	109-21-7	10.78	1.17	1.08	1.17	1.08	0.81
	Acetic acid, phenylmethyl ester	140-11-4	19.66	1.89	0.80	1.89	0.80	0.54
	Methyl salicylate	119-36-8	20.38	0.86	0.17	0.86	0.17	
	2-Propenoic acid, 3-phenyl-, methyl ester	103-26-4	24.27	0.27	0.31	0.27	0.31	0.31
	Hexadecanoic acid, methyl ester	112-39-0	25.80	0.50		0.50		
	Benzoic acid, 2-amino-, methyl ester	134-20-3	26.19	3.38	1.21	3.38	1.21	0.73
	Dimethyl phthalate	131-11-3	26.81	0.79		0.79		
	1,2-Benzenedicarboxylic acid, butyl methyl ester	117-82-8	29.08	0.29		0.29		
	Phthalic acid, isobutyl nonyl ester	28553-12-0	29.37	3.86		3.86		
	Dibutyl phthalate	84-74-2	30.91	2.27		2.27		
**Phenols**	Phenol, 3-methyl-	108-39-4	9.28		0.09			
**Ethers**	Ethanol, 2-butoxy-	111-76-2	14.42			0.91	0.65	0.50
	n-Butyl ether	142-96-1	4.28				1.03	0.78
	12-Crown-4	294-93-9					0.56	
**Acids**	Octanoic Acid	124-07-2	24.03	0.61	0.52	2.72	1.24	1.06
	Acetic acid						0.14	
	Nonanoic acid	112-05-0	25.32		0.62		1.05	0.59
	Hexanoic acid	142-62-1	21.34		0.30			0.46
	Tetradecanoic acid	544-63-8	30.91	0.57				
**Alkane**	Dodecane	112-40-3	10.30	0.28	0.12	0.26	0.43	0.24
	Tetradecane	629-59-4	14.33	0.54		0.60	0.61	0.47
	Pentadecane	629-62-9	16.04	3.97	2.83	3.24	4.47	3.92
	Hexadecane	544-76-3	17.63	0.28		0.09		
	Pentadecane, 2,6,10,14-tetramethyl-	1921-70-6	18.67	4.95	1.53	3.36	12.45	4.50
	Heptadecane	629-78-7	19.13	7.46	1.68	7.51	5.79	7.43
	Nonadecane	629-92-5	21.89	0.56				
	Oxirane, heptadecyl-						0.27	
	1-Methyl-2-methylenecyclohexane	2808-75-5	21.11	0.49	0.66			
	1,4-Octadiene	5675-25-2	21.11					0.49
	Nonane	111-84-2	3.10		0.38			
**Alkene**	Styrene	100-42-5	11.63	1.38	0.69	1.23	1.50	1.15
	1,3-Cyclooctadiene	1700-10-3	18.95	2.47	2.67	1.97		3.16
	9-Nonadecene	31035-07-1	22.14	0.33				
	3,5-Octadiene, (Z,Z)-	25001-92-7	3.61		0.19			
	1,3-trans,5-cis-octatriene	1871-52-9	7.87		0.63			
	E,Z-3-Ethylidenecyclohexene	16631-62-2	7.93		0.75			
	Cyclohexene, 3-methyl-	591-48-0	14.46		0.28			
**Aromatic Hydrocarbon**	Naphthalene	91-20-3	19.90			0.74		
	Toluene	108-88-3	6.26			0.49	0.43	
	Benzene, 1,3-dimethyl-	108-38-3	8.88	0.41		0.40	0.45	
	Ethylbenzene						0.29	
	p-Xylene	106-42-3	8.85		0.22			0.33
**Heterocyclic Compounds**	Pyridine	110-86-1	9.83	7.78	2.82	10.07	7.82	9.86
	Indole	120-72-9	28.46		0.13	0.83	0.42	0.26
	Furan, 2-ethyl-	3208-16-0	4.10		0.64			
	Furan, 2-pentyl-	3777-69-3	11.06		0.11			
	trans-2-(2-Pentenyl)furan	70424-14-5	12.55		0.68			
**Carbonyl Compound**	9-Octadecenamide, (Z)-	301-02-0	31.86				1.62	

**Sample Control:** Natural fermentation (no exogenous microorganisms introduced). **Sample CA** represents a commercially available sample. **Sample LS+PP+LC:** Inoculated with a mixed microbial solution of LS+PP+LC in a 1:1:1 ratio. **Sample LS+PP+LC+SX:** Inoculated with a mixed microbial solution of LS+PP+LC+SX in a 2:2:2:1 ratio. **Sample PP+LC:** Inoculated with a mixed microbial solution of PP+LC in a 1:1 ratio.

**Table 4:** Types and relative contents of volatile flavor compounds in five fermented dried salted Spanish mackerel (FDSSM) samples.

Category	Control		CA		LS+PP+LC		LS+PP+LC+SX		PP+LC	
	NO	Relative Content (%)	NO	Relative Content (%)	NO	Relative Content (%)	NO	Relative Content (%)	NO	Relative Content (%)
Aldehydes	7	20.97%	15	28.12%	6	9.59%	6	10.17%	6	20.15%
Alcohols	4	6.42%	6	6.69%	8	6.78%	5	8.10%	4	6.67%
Ketones	4	5.87%	4	12.96%	2	2.45%	2	1.84%	5	7.55%
Esters	2	0.80%	3	0.53%	10	15.27%	5	3.57%	4	2.38%
Phenols	0	0.00%	1	0.09%	0	0.00%	0	0.00%	0	0.00%
Ethers	0	0.00%	0	0.00%	1	0.91%	3	2.23%	2	1.27%
Acids	2	1.18%	3	1.44%	1	2.72%	3	2.43%	3	2.11%
Hydrocarbons	12	23.11%	13	12.63%	11	19.91%	10	26.69%	9	21.69%
Heterocyclic Compounds	1	7.78%	5	4.38%	2	10.90%	2	8.23%	2	10.12%
Carbonyl Compound	0	0.00%	0	0.00%	0	0.00%	1	1.62%	0	0.00%
Total	32	66.12%	51	66.84%	41	68.53%	37	64.89%	35	71.95%

**Sample Control:** Natural fermentation (no exogenous microorganisms introduced). **Sample CA** represents a commercially available sample. **Sample LS+PP+LC:** Inoculated with a mixed microbial solution of LS+PP+LC in a 1:1:1 ratio. **Sample LS+PP+LC+SX:** Inoculated with a mixed microbial solution of LS+PP+LC+SX in a 2:2:2:1 ratio. **Sample PP+LC:** Inoculated with a mixed microbial solution of PP+LC in a 1:1 ratio.

**Table 5:** Characteristic flavor compounds and ROAV of five fermented dried salted Spanish mackerel (FDSSM) samples.

Components	Detection Threshold/(μg/kg)	ROAV					Odor
		Control	CA	LS+PP+LC	LS+PP+LC+SX	PP+LC	
Hexanal	4.5	2.29	8.89	39.21		1.01	Pine, turpentine
Heptanal	3.0	1.32	5.53	24.83	2.77	0.72	Fatty, almond
Octanal	0.7	6.10	45.95	100.00	9.90	3.09	Fatty, lemon
Nonanal	1.0	2.95	21.61	61.79	6.21	1.39	Fatty, citrus
Benzaldehyde	350.0	0.02	0.03	0.28	0.02	0.01	Sharp, sweet
Butanal, 3-methyl-	0.2	100.00	100.00		100.00	100.00	Fruity, almond
4-Heptenal, (Z)-	0.8		10.33				Green, linseed oil-like, vegetal aroma
2,4-Heptadienal, (E,E)-	10.0		1.05				Green flavor, green, fatty, Rancid odor, fishy
2-Nonenal, (E)-	0.08		24.27				Watermelon-like, fishy
2-Penten-1-ol, (Z)-	89.2	0.04	0.13	0.80	0.09	0.04	Green, rubber-like odor
1-Octen-3-ol	10.0	0.51	1.89	5.92	0.87	0.31	Mushroom, raw
1-Heptanol	3.0	0.85	1.96	10.77	1.09	0.58	Musty, sweet
2,3-Octanedione	2.52	1.53				0.67	Fruity
2-Nonanone	5.0	0.12	1.31			0.07	Blue cheese aroma, winey
Toluene	200.0			0.13	0.01		Plastic-like odor, chemical-like odor, petrol odor
Pyridine	2000.0	0.01	0.01	0.26	0.02	0.01	Pungent, fish-like aroma, Amine-like odor
Indole	140.0		0.01	0.31	0.01	0.00	Floral
Furan, 2-ethyl-	2.3		1.85				Beany, malty

**Sample Control:** Natural fermentation (no exogenous microorganisms introduced). **Sample CA** represents a commercially available sample. **Sample LS+PP+LC:** Inoculated with a mixed microbial solution of LS+PP+LC in a 1:1:1 ratio. **Sample LS+PP+LC+SX:** Inoculated with a mixed microbial solution of LS+PP+LC+SX in a 2:2:2:1 ratio. **Sample PP+LC:** Inoculated with a mixed microbial solution of PP+LC in a 1:1 ratio.

**Table 6:** Relative cell viabilities of freeze-dried strains in the presence of combinations of cryoprotectants.

No.	15% Inulin	15% Arabic Gum	5% Skim Milk Powder	5% Sodium Glutamate	Freeze-Drying Survival Rate (%)
1	12.50%	12.50%	12.50%	62.50%	69.86 ± 0.23 ^k^
2	0.00%	100.00%	0.00%	0.00%	5.56 ± 0.25 ^b^
3	0.00%	0.00%	50.00%	50.00%	60.9 ± 0.18 ^k^
4	33.33%	33.33%	33.33%	0.00%	31.99 ± 0.02 ^h^
5	50.00%	0.00%	0.00%	50.00%	55.64 ± 0.25 ^j^
6	50.00%	50.00%	0.00%	0.00%	12.67 ± 0.45 ^e^
7	25.00%	25.00%	25.00%	25.00%	18.55 ± 0.12 ^f^
8	0.00%	33.33%	33.33%	33.33%	31.22 ± 0.14 ^h^
9	50.00%	0.00%	50.00%	0.00%	7.88 ± 0.07 ^c^
10	62.50%	12.50%	12.50%	12.50%	38.95 ± 0.09
11	0.00%	0.00%	100.00%	0.00%	57.65 ± 0.23 ^j^
12	100.00%	0.00%	0.00%	0.00%	46.06 ± 0.19 ^i^
13	0.00%	50.00%	50.00%	0.00%	33.69 ± 0.11 ^h^
14	0.00%	50.00%	0.00%	50.00%	12.52 ± 0.05 ^e^
15	12.50%	62.50%	12.50%	12.50%	26.43 ± 0.22 ^g^
16	33.33%	33.33%	0.00%	33.33%	57.19 ± 0.54 ^j^
17	33.33%	0.00%	33.33%	33.33%	41.89 ± 0.37 ^i^
18	0.00%	0.00%	0.00%	100.00%	10.51 ± 0.17 ^d^
19	12.50%	12.50%	62.50%	12.50%	31.53 ± 0.34 ^h^
20	0.00%	0.00%	0.00%	0.00%	0.77 ± 0.03 ^a^

Values are presented as mean ± SD (n = 3). Within the same column, different superscript letters (a–k) denote statistically significant differences among groups (*p* < 0.05).

## Data Availability

The original contributions presented in this study are included in the article/Supplementary Materials. Further inquiries can be directed to the corresponding authors.

## Supplementary Materials

The following supporting information can be downloaded at https://www.mdpi.com/article/10.3390/foods15162862/s1, Table S1: Box–Behnken experimental design and results; Table S2: Analysis of variance of sensory score regression model; Table S3: Relative cell viabilities of freeze-dried strains (%) in the presence of different protectants; Figure S1: Sample image of CA, DVSS and LS+PP+LC.
