# Supplementary material for: Development of Fermented Dried Salted Spanish Mackerel and Analysis of Its Flavor Compounds, Fermentation Conditions, and Preparation of a Directed Vat-Set Starter
Source: Foods. 2026 Aug 17;15(16):2862. doi: 10.3390/foods15162862 (PMC13512149; doi:10.3390/foods15162862)
Supplement: Supplementary file 1 [file foods-15-02862-s001.zip › foods-4469794-supplementary.pdf]

Supplementary materials

Supplementary Table S1 Box-Behnken experimental design and results

NO	Fermentation time (h)	Fermentation temperature (℃)	Strain addition amount (%)	Sensory Scores
1	1.000	0.000	-1.000	85.5
2	0.000	-1.000	1.000	84.3
3	1.000	1.000	0.000	81.2
4	-1.000	-1.000	0.000	72.5
5	1.000	0.000	1.000	87.8
6	1.000	-1.000	0.000	82.0
7	0.000	1.000	1.000	76.7
8	0.000	-1.000	-1.000	85.4
9	-1.000	1.000	0.000	87.0
10	-1.000	0.000	-1.000	88.5
11	0.000	0.000	0.000	90.0
12	0.000	0.000	0.000	87.5
13	0.000	1.000	-1.000	87.5
14	0.000	0.000	0.000	92.8
15	-1.000	0.000	1.000	84.6
16	0.000	0.000	0.000	92.1
17	0.000	0.000	0.000	91.0

Supplementary Table S2. Analysis of variance of sensory score regression model

Source	Sum of Squares	df	Mean Square	F-value	P-value	Significance
Model	388.75	9	43.19	3.93	0.0488	*
A	1.53	1	1.53	0.14	0.6920	
B	7.03	1	7.03	0.64	0.4141	
C	28.12	1	28.12	2.56	0.1960	
A^2	52.69	1	52.69	4.79	0.0724	
B^2	179.95	1	179.95	16.36	0.0050	*
C^2	3.51	1	3.51	0.32	0.7058	
AB	60.06	1	60.06	5.46	0.0557	
AC	9.00	1	9.00	0.82	0.3841	
BC	30.25	1	30.25	2.75	0.1897	
Residual	78.06	7	11.15			
Lack of Fit	60.87	3	20.29	4.72	0.0839	
Pure Error	17.19	4	4.30			
Cor Total	450.84	16				
	R^2=0.8269	R^2Adj=0.6042		S/N=5.990		

Note： * indicates *P≤0.05,*** indicates *P≤0.01*

Supplementary Table S3. Relative cell viabilities of freeze dried strains (%) in the presence of different protectants

concentration	normal saline	sucrose	trehalose	inulin	dextrin	arabic gum	skim milk powder	sodium glutamate	glycerol	Vc
5%	0.00	0.00	0.00	4.52	3.26	3.44	18.82	40.18	0.00	0.18
15%	0.00	0.00	3.80	9.95	0.54	23.71	14.12	5.07	9.05	0.00
30%	0.00	0.05	6.52	4.34	0.18	2.53	22.44	14.12	0.91	0.72

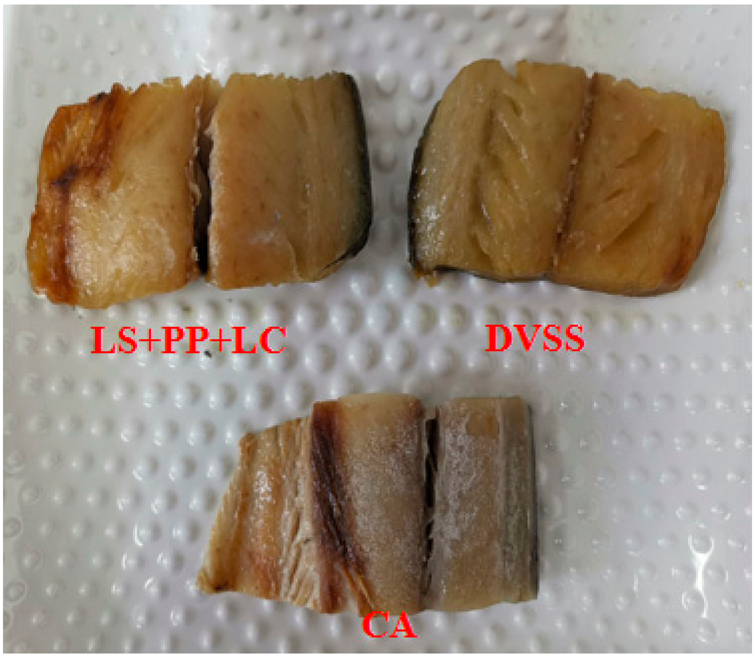

Supplementary Figure S1. Sample Image of CA,DVSS and LS+PP+LC

Sample CA represents a commercially available sample.**Sample DVSS:** Inoculated with directed vat set starter. **Sample LS+PP+LC:** Inoculated with a mixed microbial solution of LS+PP+LC in a 1:1:1 ratio.
